# The Hidden Link: Cryoglobulinemic Membranoproliferative Glomerulonephritis Secondary to Monoclonal Gammopathy of Renal Significance

**DOI:** 10.14740/jmc5318

**Published:** 2026-07-28

**Authors:** Samar Mehyar, Amanda Gomes, Harneet K. Ghumman, Avneet K. Ghumman, Noor Eweis, Syedehmaryam Pishva, Jerry Kenmoe

**Affiliations:** a Department of Internal Medicine, McLaren Bay Region Hospital, Bay City, MI, USA; b Department of Nephrology, McLaren Bay Region Hospital, Bay City, MI, USA; c Department of Internal Medicine, McLaren Health Care/Michigan State University, Flint City, MI, USA; d Hematology and Medical Oncology Department, McLaren Greater Lansing, Lansing City, MI, USA

**Keywords:** Membranoproliferative, Glomerulonephritis, MPGN, MGRS, MGUS

## Abstract

Membranoproliferative glomerulonephritis (MPGN) is a histopathological pattern that represents a spectrum of glomerular injury diseases. MPGN is usually associated with infections, autoimmune diseases and monoclonal gammopathies. Monoclonal gammopathy of renal significance (MGRS) is any proliferative B-cell or plasma-cell disease that causes abnormal buildup of monoclonal proteins in the kidneys. It is usually underdiagnosed due to its rarity. We present a case of a 74-year-old woman referred to nephrology with worsening creatinine levels, proteinuria and microscopic hematuria. Laboratory evaluation showed low C3 levels and elevated serum free light chain ratio. Kidney biopsy showed immune deposits consistent with cryoglobulinemic glomerulonephritis secondary to MGRS. Patient was treated with prednisone and mycophenolate mofetil, resulting in improvement in the creatinine functions, proteinuria, and hematuria.

## Introduction

Membranoproliferative glomerulonephritis (MPGN) is a glomerular injury that is characterized by endocapillary proliferation and mesangial deposits of immune complexes [1]. MPGN usually presents as nephritic syndrome. MPGN is subclassified as immune complex MPGN (IC-MPGN) and complement 3 glomerulopathy (C3G). IC-MPGN can occur secondary to infections, autoimmune diseases, paraproteinemia and malignancy [2]. Cryoglobulinemic MPGN is most associated with infections, specifically hepatitis B and C virus [3, 4].

MPGN is a rare glomerular disease and one of the less common causes of nephrotic syndrome. Its incidence has decreased over the decades, and it currently accounts for around two cases per million population per year. It has variable and unusual clinical presentations from being asymptomatic to hematuria. It can be primary (idiopathic), and secondary to various causes such as infections, autoimmune diseases, and tumors [5]. Cryoglobulinemia is a rare medical condition, it is characterized by immunoglobulins that deposit at lower temperatures in the small and medium-sized blood vessels causing endothelial injury and organ damage [6]. We present a case of a 74-year-old woman who was initially diagnosed with monoclonal gammopathy of unknown significance (MGUS) after initial serology and bone marrow biopsy. Her creatinine levels continued to spike up until a computed tomography (CT)-guided biopsy of the kidney confirmed a mixed cryoglobulinemic glomerulonephritis. This is a unique case, as the patient was known to have an immunoglobulin (Ig)G kappa paraprotein and was initially diagnosed with MGUS following bone marrow biopsy. MGUS is considered a premalignant condition that can evolve to multiple myeloma (MM) in some patients. Moreover, MGUS is common in patients with chronic kidney disease (CKD) and is more prevalent in older patients with CKD. The diagnosis of monoclonal gammopathy of renal significance (MGRS) by kidney biopsy is important for guiding treatment and improving renal prognosis.

## Case Report

A74-year-old woman with past medical history of stable CKD stage III attributed to hypertension, nonischemic cardiomyopathy, and paroxysmal atrial fibrillation was referred to the nephrology because of worsening creatinine. She was initially found to have microscopic hematuria and proteinuria during a previous hospital stay for *Clostridium difficile* infection. In March 2021, during a workup for post herpetic neuralgia by neurology, the patient was found to have an IgG kappa paraprotein; however, she was not evaluated by hematology at that time. The patient was referred to nephrology in June 2022 with microscopic hematuria, proteinuria, and worsening creatinine levels (from 1 to 1.7 mg/dL). At that time, proteinuria was quantified at 0.4 mg/g, urine microscopy showed persistent microscopic hematuria with blood urine of 250 (Tables 1, 2). Serum protein electrophoresis (SPEP) showed elevated kappa free light chain 10.37 mg/L (normal range is 3.3–19.4 mg/L). Lambda free light chain was 1.75 mg/L (normal range is 5.7–26.3 mg/L), kappa/lambda free light chain ratio was 5.93 (normal range is 0.26–1.65). Serum immunoelectrophoresis showed IgM kappa level of 0.3 g/dL (normal range is 3.3–19.4 mg/L), and IgG Kappa level of 0.45 g/dL (normal range is 3.3–19.4 mg/L) (Table 3). Urine protein electrophoresis (UPEP) was negative. Results were remarkable for a low C3 level of 77 mg/dL. Further laboratory workup was negative for cryoglobulin, antinuclear antibodies (ANA), myeloperoxidase–proteinase 3 (MPO/PR3), anti–glomerular basement membrane (anti-GBM) antibodies, C4, hepatitis B and C, human immunodeficiency virus (HIV), anti-Ro, and anti-La levels (Table 4). Patient was referred to oncology for further evaluation and bone marrow biopsy.

**Table 1:** CBC, CMP and Iron Studies

Serum	Patient values	Reference range
WBC count	4.87	4,000–11,000
Hemoglobin	11.5	12.0–15.5 g/dL
Platelet count	303	150,000–450,000/µL
Anion gap	10.5	4–12 mEq/L
Sodium	141	135–145 mEq/L
Potassium	5.2	3.6–5.2 mEq/L
Chloride	105	96-106 mEq/L
CO_2_	25.4	23–29 mmol/L
Calcium level	9.8	8.5–10.5 mg/dL
BUN	35.6	6–20 mg/dL
Creatinine	1.7	0.5–1.1 mg/dL
Glucose	63	70–99 mg/dL
Protein	6.8	6.0–8.3 g/dL
Albumin	4.4	3.4–5.4 g/dL
Globulin	2.5	2.0–3.5 g/dL
ALT	19	4–36 U/L
AST	22	8–33 U/L
Alkaline phosphatase	84	44–147 IU/L
Bilirubin total	0.9	0.1–1.2 mg/dL
eGFR	33	> 90
Ferritin	95.9	30–400 ng/mL
Iron	63	50–170 µg/dL
TIBC	300	149–492 µg/dL
Transferrin	214	250–450 µg/dL

CBC: complete blood count; CMP: comprehensive metabolic panel; ALT: alanine aminotransferase; AST: aspartate aminotransferase; eGFR: estimated glomerular filtration rate; TIBC: total iron-binding capacity; BUN: blood urea nitrogen; WBC: white blood cell.

**Table 2:** Urine Studies

Urine	Patient values	Reference range
UA color	Yellow	Clear to pale yellow
UA appearance	Clear	Clear
UA PH	5	4.6–8.0
UA specific gravity	1.013	1.005–1.030
UA bilirubin	Negative	Negative
UA blood	Trace	0–2 RBCs/HPF
UA glucose	250	0–0.8 mmol/L
UA ketones	Negative	< 0.6 mmol/L
UA leukocyte esterase	Trace	Negative
UA nitrite	Negative	Negative
UA protein	Trace	150 mg/day
UA urobilinogen	0.2	UA urobilinogen
Urine creatinine	60.1	0.8–2.0 g/day
Urine microalbumin/creatinine ratio	191	< below 30 mg/g
Urine microalbumin	11.5	< 30 mg/day
Urine protein	23.2	< 150 mg/day
Urine protein/creatinine ratio	0.38	< 0.2 mg/mg

UA: urinalysis; RBC: red blood cell; HPF: high-power field.

**Table 3:** Protein Electrophoresis

Serum	Patient values	Reference range
Kappa free light chain	10.37	3.3–19.4 mg/L
Lambda free light chain	1.75	5.7–26.3 mg/L
Kappa/lambda FLC ratio	5.93	0.26–1.65
Calculated albumin	4.28	3.5–5.0 g/dL
Urine protein electrophoresis	No Bence jones proteins	Albumin

**Table 4:** Autoimmune Workup

Serum	Patient values	Reference range
ANA screen	Negative	Negative
Glomerular basement membrane IgG	Negative	< 7.0 U/mL
Myeloperoxidase antibody	Negative	< 0.4 U
Proteinase 3	Negative	< 2 IU/mL
Quantitative rheumatoid arthritis factor	< 10	< 14 IU/mL
C3 complement	76.8	88–201 mg/dL
C4 complement	13.7	15–45 mg/dL
IgA	82.9	70–400 mg/dL
IgG	1,042.00	600–1,600 mg/dL
IgM	333	40–250 mg/dL

ANA: antinuclear antibody; Ig: immunoglobulin.

Bone marrow biopsy showed a normocellular to slightly hypocellular marrow (10–20% cellular for age) with trilineage hematopoiesis. A single small lymphoid aggregate was noted. No large clusters of plasma cells were identified. CD138 highlighted approximately 1–2% plasma cells. Flow cytometry demonstrated reticulin fibrosis with occasional lymphoid aggregates in the bone marrow. Congo red staining was negative for amyloidosis. The bone scan was unremarkable. A second bone marrow aspirate and biopsy done a couple of months later showed a 10–20% cellular, small kappa restricted clonal B-cell population by flow, and CG 46,XX. MM fluorescence *in situ* hybridization (FISH) panel was normal. There was no increase in blasts (more than 1%), and the plasma cells count was 1%, with no definite increase in plasma cells or reticulin fibrosis.

**Figure 1:**
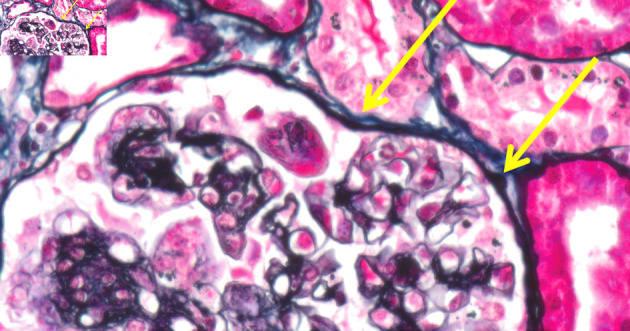
Intraluminal cryoglobulin deposits (yellow arrows).

**Figure 2:**
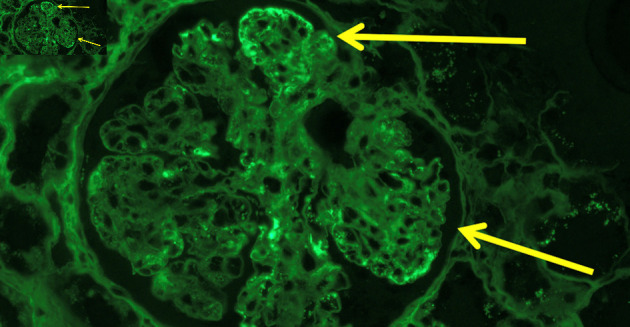
IgG immunofluorescence (yellow arrows) indicating cryoglobulinemic glomerulonephritis. Ig: immunoglobulin.

**Figure 3:**
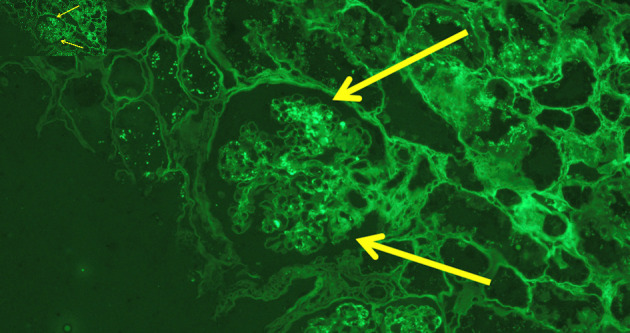
Immunofluorescence staining showing IgM deposition (yellow arrows). Ig: immunoglobulin.

**Figure 4:**
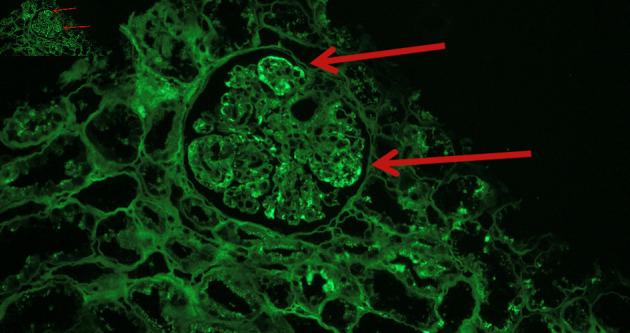
Immunofluorescence stain showing kappa light chain deposits (red arrows).

**Figure 5:**
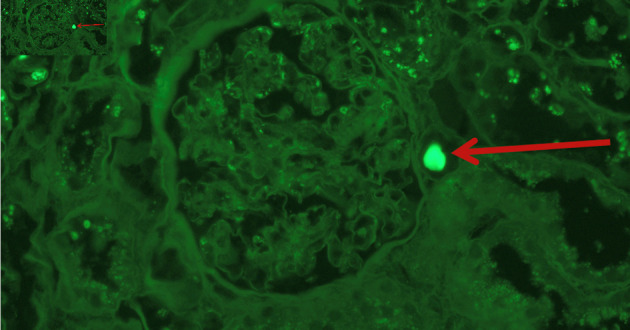
Immunofluorescence stain kappa deposits (red arrow).

**Figure 6:**
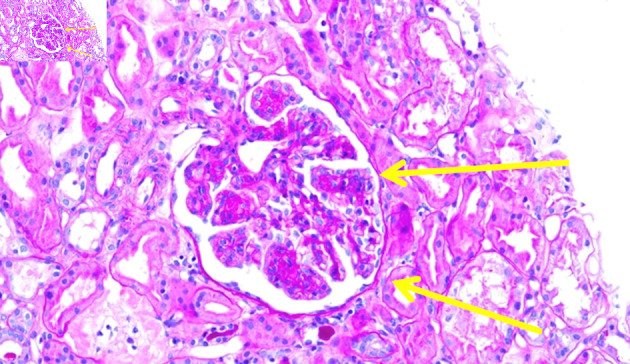
MPGN pattern on periodic acid Schiff (yellow arrows).

The patient underwent a CT-guided left kidney biopsy, which showed a membranoproliferative pattern of glomerular injury with masked immune deposits overlying glomerulonephritis and kappa and lesser IgG and lambda light chains (Figs. 1–6), most consistent with mixed cryoglobulinemic glomerulonephritis. Initially, the patient was monitored conservatively; however, in the spring of 2024, the patient’s urine protein-to-creatinine ratio (UPC) increased to 1.4 g, prompting initiation of intervention. The patient was started on oral prednisone 40 mg daily with tapering, mycophenolate mofetil (MMF) and prophylaxis for *Pneumocystis jirovecii* pneumonia (PJP), resulting in improvement in proteinuria and serum creatinine. Patient has successfully remained on MMF with a stable serum creatinine level of 1.4 mg/dL. The UPC has decreased to 0.2 mg/g, with resolution of microscopic hematuria.

## Discussion

This case involves a woman presented with worsening proteinuria, microscopic hematuria, and increasing creatinine levels. Laboratory evaluation showed a low C3 level and negative results for hepatitis B and C, autoimmune and cryoglobulin levels. SPEP revealed increase in the kappa, lambda, and kappa/lambda ratio. Bone marrow biopsy findings were suggestive of MGUS. A repeated kidney biopsy was performed to confirm the diagnosis of MGRS and guide appropriate treatment. Patient ultimately required immunosuppression for MPGN with prednisone followed by a taper and MMF, with subsequent stabilization of the renal function and proteinuria and resolution of microscopic hematuria.

MPGN is a rare condition characterized by mesangial hypercellularity and subendothelial deposition of the complements or immune complexes causing glomerular wall thickening [5]. It accounts for about 7–10% of all glomerulonephritis cases [5]. MPGN in older patients is commonly associated with cryoglobulinemia and hepatitis infections [7].

Recent classification of MPGN is based on immunofluorescence examination findings. It can be subdivided into immune complex-mediated subtype, complement-mediated subtype, and a subtype without immune complexes or complements. Immune complex-mediated subtype is associated with chronic antigenemia with or without the presence of complement levels. It is mainly caused by infections, most commonly hepatitis B and C, autoimmune diseases such as systemic lupus erythematosus (SLE) and Sjogren’s, monoclonal gammopathy, or idiopathic [8].

According to the 2021–2022 International Kidney and Monoclonal Gammopathy Research Group (IKMG) consensus, MGRS is defined as any proliferative B-cell or plasma-cell disorder that produces nephrotoxic monoclonal immunoglobulins [9]. MGRS can be caused by a variety of kidney diseases including MGUS, amyloidosis and proliferative glomerulonephritis with monoclonal immunoglobulin deposits (PGNMID). A renal biopsy should always be done whenever MGRS is suspected [10].

MGUS has a prevalence of approximately 3% in people over 50 years of age, 5% in those over 70, and 8% in those over 80 years of age. As CKD is more common in patients over 60 years of age, patients with MGUS can also coincidentally have CKD. In old patients with MGUS and renal manifestations such as elevated creatinine, hematuria or proteinuria, performing renal biopsy is crucial to avoid missing a diagnosis of MGRS kidney disorders, which is more likely to occur in old patients [10].

MGRS does not meet the criteria for overt MM but is associated with significant mortality, depending on the severity of the monoclonal immunoglobulin deposition in the kidneys and other systemic organs. Renal failure is a common presenting symptom, with or without microscopic hematuria [11]. Current treatment of MGRS associated with MPGN depends on the symptoms and the degree of B-cell proliferation. In patients who are asymptomatic or have mild symptoms and low-grade proliferation, observation is recommended. However, patients with progressive symptoms may require further treatment [11].

MPGN is usually caused by infections such hepatitis C virus, autoimmune diseases, and less commonly, MGRS. It is common in older patients with CKD [11, 12]. The prevalence of MPGN in patients with MGUS is underrecognized and understudied due to rarity of the disease, and more research is needed to better understand the association between both. Kidney biopsy is crucial whenever MGRS is suspected, it should be undertaken in patients with a paraprotein, elevated creatinine levels, hematuria or proteinuria. This is important to assess severity and determine treatment, especially in older patients with negative serological workup for infectious and autoimmune diseases. This case shows the importance of immunosuppressive therapy over clinical observation in symptomatic patients for the management of the disease.

### Conclusions

MPGN is a pattern of glomerular injury that is characterized by subendothelial and mesangial deposition of immune complexes. It is usually caused by infections such as hepatitis C virus infection, autoimmune disease, and less commonly from MGRS. Kidney biopsy is important to confirm the diagnosis, assess its severity, and determine the type of treatment. Further studies are still needed to acknowledge the prevalence of MPGN in patients with MGUS to start the appropriate treatment in a timely manner.

## Data Availability

All data supporting the findings of this case report are included within the manuscript. Additional information is available from the corresponding author upon reasonable request.
